# Supplementary material for: Altered Cerebral Blood Flow in the Progression of Chronic Kidney Disease
Source: J Pers Med. 2023 Jan 11;13(1):142. doi: 10.3390/jpm13010142 (PMC9863421; doi:10.3390/jpm13010142)
Supplement: Supplementary file 1 [file jpm-13-00142-s001.zip › jpm-2091649-supplementary.pdf]

CBF values in the right insula and left hippocampus in CKD and HC

Group	right_insula	left_hippocampus
HC	.34	-.40
HC	.82	-.52
HC	.31	-.41
HC	.62	-.39
HC	.64	-.84
HC	.81	-.35
HC	.91	-.29
HC	.60	-.16
HC	.65	-.45
HC	.68	-.34
HC	.19	-.64
HC	.26	-.29
HC	.19	-.42
HC	.14	-.69
HC	.43	-.67
HC	.28	-.38
HC	.56	-1.00
HC	.61	-.14
HC	.40	-.38
HC	.60	-.04

HC	.70	.09
HC	.43	-.35
HC	.55	-.25
HC	.64	-.58
HC	.29	-.54
HC	.33	-.60
HC	.46	-.38
HC	.24	-.11
HC	.62	-.19
HC	.07	-.52
HC	.65	-.28
CKD4	.21	-.10
CKD4	.11	-.14
CKD4	.42	-.23
CKD4	.17	-.20
CKD4	.19	-.15
CKD4	-.68	.23
CKD4	.05	.09
CKD4	.05	-.36
CKD4	.29	.01
CKD4	.40	.36
CKD4	.43	-.62

CKD4	.07	.10
CKD4	-.21	.80
CKD4	.04	.12
CKD4	-.13	-.27
CKD4	.36	-.19
CKD4	.56	.65
CKD4	.15	1.44
CKD4	-.26	-.27

The coordinate points of the ROC curve, and the corresponding sensitivity, 1-specificity.

Coordinates of the Curve

Test Result Variable(s): left_hippocampus

Positive if Greater Thanor Equal To[a]	Sensitivity	1 - Specificity
-2.0027	1.000	1.000
-0.9190	1.000	0.968
-0.7650	1.000	0.935
-0.6827	1.000	0.903
-0.6554	1.000	0.871
-0.6308	1.000	0.839

-0.6131	0.947	0.839
-0.5924	0.947	0.806
-0.5618	0.947	0.774
-0.5340	0.947	0.742
-0.5218	0.947	0.710
-0.4866	0.947	0.677
-0.4395	0.947	0.645
-0.4198	0.947	0.613
-0.4061	0.947	0.581
-0.3931	0.947	0.548
-0.3843	0.947	0.516
-0.3789	0.947	0.484
-0.3771	0.947	0.452
-0.3680	0.947	0.419
-0.3569	0.895	0.419
-0.3498	0.895	0.387
-0.3411	0.895	0.355
-0.3148	0.895	0.323
-0.2926	0.895	0.290
-0.2844	0.895	0.258
-0.2736	0.895	0.226
-0.2687	0.842	0.226
-0.2597	0.789	0.226
-0.2428	0.789	0.194
-0.2159	0.737	0.194
-0.1966	0.684	0.194
-0.1912	0.684	0.161
-0.1763	0.632	0.161
-0.1567	0.632	0.129
-0.1460	0.579	0.129
-0.1428	0.526	0.129
-0.1250	0.526	0.097
-0.1053	0.526	0.065
-0.0725	0.474	0.065
-0.0167	0.474	0.032
0.0473	0.421	0.032
0.0886	0.421	0.000
0.0961	0.368	0.000
0.1100	0.316	0.000
0.1765	0.263	0.000
0.2975	0.211	0.000
0.5043	0.158	0.000
0.7236	0.105	0.000

1.1215	0.053	0.000
2.4432	0.000	0.000

Coordinates of the Curve

Test Result Variable(s): right_insula

Positive if Greater Thanor Equal To[a]	Sensitivity	1 - Specificity
-1.6790	0.000	0.000
-0.4712	0.053	0.000
-0.2367	0.105	0.000
-0.1677	0.158	0.000
-0.0427	0.211	0.000
0.0439	0.263	0.000
0.0484	0.316	0.000
0.0582	0.368	0.000
0.0678	0.421	0.000
0.0891	0.421	0.032
0.1234	0.474	0.032
0.1451	0.474	0.065
0.1612	0.526	0.065
0.1781	0.579	0.065
0.1889	0.632	0.065
0.1923	0.632	0.097
0.2013	0.632	0.129
0.2257	0.684	0.129
0.2533	0.684	0.161
0.2741	0.684	0.194
0.2868	0.684	0.226
0.2911	0.684	0.258
0.3025	0.737	0.258
0.3206	0.737	0.290
0.3344	0.737	0.323

0.3488	0.737	0.355
0.3788	0.789	0.355
0.4005	0.842	0.355
0.4086	0.842	0.387
0.4221	0.895	0.387
0.4296	0.947	0.387
0.4325	0.947	0.419
0.4493	0.947	0.452
0.5052	0.947	0.484
0.5519	0.947	0.516
0.5588	0.947	0.548
0.5804	1.000	0.548
0.6007	1.000	0.581
0.6057	1.000	0.613
0.6134	1.000	0.645
0.6182	1.000	0.677
0.6315	1.000	0.710
0.6437	1.000	0.742
0.6474	1.000	0.774
0.6510	1.000	0.806
0.6660	1.000	0.839
0.6891	1.000	0.871
0.7544	1.000	0.903
0.8165	1.000	0.935
0.8677	1.000	0.968
1.9132	1.000	1.000

Sample size analysis

Through power analysis of the three neurocognitive tests, MOCA, MMSE, and DST, which were significantly different, the required sample sizes were calculated to be 33 for the HC group and 20 for the CKD4 group, which is close to the number of subjects collected in our study; 43 for the HC group and 26 for the CKD4 group for MMSE; and 24 for the HC group and 15 for the CKD4 group for DST.
